# Emergence of a novel bovine spongiform encephalopathy (BSE) prion from an atypical H-type BSE

**DOI:** 10.1038/srep22753

**Published:** 2016-03-07

**Authors:** Kentaro Masujin, Hiroyuki Okada, Kohtaro Miyazawa, Yuichi Matsuura, Morikazu Imamura, Yoshifumi Iwamaru, Yuichi Murayama, Takashi Yokoyama

**Affiliations:** 1 Influenza and Prion Disease Research Center, National Institute of Animal Health (NIAH), National Agriculture Food Research Organization (NARO), 3-1-5 Kannondai, Tsukuba, Ibaraki 305-0856, Japan

## Abstract

The H-type of atypical bovine spongiform encephalopathy (H-BSE) was serially passaged in bovinized transgenic (TgBoPrP) mice. At the fourth passage, most challenged mice showed a typical H-BSE phenotype with incubation periods of 223 ± 7.8 days. However, a different phenotype of BSE prion with shorter incubation periods of 109 ± 4 days emerged in a minor subset of the inoculated mice. The latter showed distinct clinical signs, brain pathology, and abnormal prion protein profiles as compared to H-BSE and other known BSE strains in mice. This novel prion was transmitted intracerebrally to cattle, with incubation periods of 14.8 ± 1.5 months, with phenotypes that differed from those of other bovine prion strains. These data suggest that intraspecies transmission of H-BSE in cattle allows the emergence of a novel BSE strain. Therefore, the continuation of feed ban programs may be necessary to exclude the recycling of H-BSE prions, which appear to arise spontaneously, in livestock. Such measures should help to reduce the risks from both novel and known strains of BSE.

Prions cause transmissible spongiform encephalopathies (TSEs), which are characterized by a spongiform change in the central nervous system and the accumulation of an abnormal prion protein (PrP^Sc^). PrP^Sc^ is a disease-associated isoform of the host-encoded prion protein^1^, and the conversion of the normal isoform (PrP^C^) to PrP^Sc^ is thought represent a central event in prion pathogenesis. PrP^Sc^ has been recognized as the major component of prions^2^, and variations in PrP^Sc^ are associated with different prion strains that, in turn, cause distinct disease phenotypes^3^.

Bovine spongiform encephalopathy (BSE) is a TSE of cattle. Most BSE cases show a unique phenotype that is thought to be caused by a single prion strain^4^. However, other atypical neuropathological and molecular phenotypes of BSEs (atypical BSEs) have been identified in aged animals^5^,^6^. Atypical BSEs have been classified into two groups, L-BSE and H-BSE, based on their biological and biochemical features^5^,^6^, which differ from those of classical BSE (C-BSE).

The worldwide occurrence of BSE is declining, primarily because of effective feed ban programs^6^. The emergence of atypical BSE cases has raised questions as to whether additional and/or modified control measures might be needed. Thus, characterization of atypical BSE prions is necessary for risk evaluation.

H-BSE was first reported in France^7^, and has been subsequently detected in several European countries and North America^5^,^6^. In one of two H-BSE cases detected in the U.S., a prion protein (PrP) amino-acid substitution that is associated with familial Creutzfeldt–Jakob disease in humans has been reported. This suggested the possibility that H-BSE might occur spontaneously or sporadically^8^. Regardless of its origin, previous studies have shown that H-BSE is transmissible to cattle^9^,^10^,^11^,^12^, as well as to wild type^13^,^14^, bovinized, and ovinized PrP transgenic mice that express murine, bovine, or ovine PrP^C^, respectively^15^,^16^,^17^. Those studies also revealed different characteristics of H-BSE relative to C-BSE and L-BSE.

Serial passages of H-BSE in wild type^18^,^19^ and bovinized PrP transgenic mice^20^ can lead to the emergence of a C-BSE-like phenotype. This suggests that H-BSE-affected cattle harbor heterogeneous prions and that structural variants of H-BSE might generate a C-BSE-like phenotype. This, in turn, raises the possibility that C-BSE originated from H-BSE. To attempt to clarify the origin of C-BSE, we serially passaged H-BSE in bovinized PrP transgenic (TgBoPrP) mice. In contrast to previous studies, we did not detect C-BSE-like prions during serial passages. However, a novel BSE prion—different from C-BSE, L-BSE, and H-BSE prions—was detected in a subset of inoculated mice. When this novel prion was inoculated intracerebrally into cattle, a novel BSE phenotype was confirmed. This study suggests that a novel BSE emerges during intraspecies transmission of H-BSE in cattle.

## Results

### Serial transmission of H-BSE in TgBoPrP mice

The results of serial transmission of the H-BSE isolate in TgBoPrP mice are shown in Table 1. All the H-BSE challenged mice developed progressive neurological disease with the incubation periods of 320.1 ± 12.2 days at primary passage. H-BSE-affected animals showed a distinctive clinical sign, namely, constant chewing of the bedding, as reported previously^16^. The incubation periods of the second and third passages were 226.9 ± 4.2 and 215.6 ± 5.0 days, respectively (Table 1)^16^. No clear differences were observed in their clinical signs, the banding pattern of PrP^Sc^, and histopathological features from the primary to third passage mice. At the fourth passage, mice from a single experimental group (#3), out of eight experiments, showed shorter incubation periods (108.8 ± 4.0 days) than the other groups. This group was challenged with brain homogenates of a mouse with 221-day incubation period. Group #3 animals showed weight loss, but no constant chewing of the bedding. This short incubation-type of BSE was designed BSE-SW (short incubation with weight loss) strain. BSE-SW was transmitted to TgBoPrP mice with 97.3 ± 3.7 day incubation periods, and their clinical signs were identical to those of mice in the experimental group #3. The other mice in the fourth passage groups showed the symptomatic chewing of the bedding, and their incubation periods were 223.3 ± 7.8 days (Table 1).

### Molecular features of PrPcore of BSE-SW

**Figure 1:**
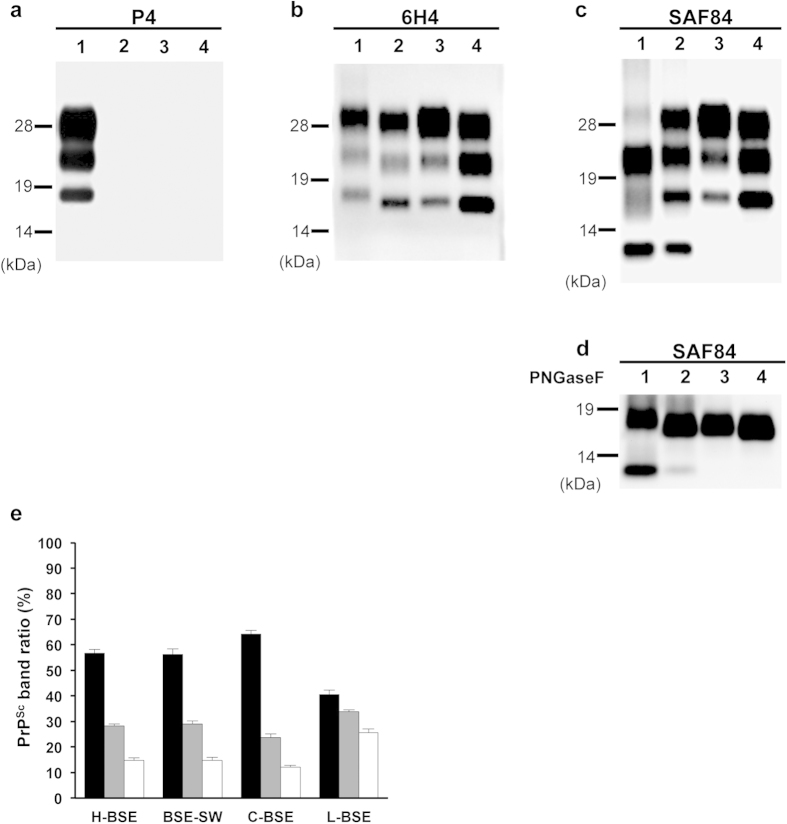
Western blot analysis of PrPcore. PrPcore from BSE prion-affected TgBoPrP mice was detected using mAbs P4 (**a**), 6H4 (**b**), and SAF84 (**c**). Lane 1, H-BSE; Lane 2, BSE-SW; Lane 3, C-BSE; Lane 4, L-BSE. All the samples were digested with 50 μg/ml PK at 37 °C for 1 h. Digested aliquots were treated with PNGaseF and probed with mAb SAF84 (**d**). Equivalents of 0.125 mg brain tissue were loaded. PrPcore of BSE-SW showed a different reaction with mAbs compared to H-BSE, as well as a distinct band pattern and molecular weight of PrPcore fragments. Molecular markers are shown on the left (kDa). Glycoform profile of PrPcore is given (**e**). Relative amounts of diglycosylated (solid black bar), monoglycosylated (gray bar), and unglycosylated (clear bar) forms of PrPcore from BSE-affected TgBoPrP mice. The signals were detected using mAb 6H4, and the results are presented as mean ± standard deviation of nine experiments. No significant differences were observed between H-BSE and BSE-SW in the glycoform ratio of PrPcore.

Western blot analysis with monoclonal antibody (mAb) 6H4 revealed that the molecular mass of proteinase K (PK) digested PrP^Sc^ (PrPcore) of BSE-SW was lower than that of H-BSE, but similar to C-BSE (Fig. 1b). MAb P4, which recognizes the N-terminal region of PrPcore, reacted with H-BSE but not with BSE-SW or C-BSE (Fig. 1a). This revealed a partial similarity between PrP^Sc^ of BSE-SW and C-BSE. In contrast, truncated 12-kDa fragments (consistent with PrP 157/163–231^14^) were observed for H-BSE and BSE-SW (Fig. 1c). Deglycosylation analysis confirmed these results. Unglycosylated PrPcore with a molecular weight of approximately 17 kDa (PrPcore #1^14^) was detected in C-BSE and BSE-SW, whereas a slightly higher molecular weight (approximately 19 kDa) of PrPcore #1 was detected in H-BSE. Unglycosylated PrPcore with a molecular weight of 12 kDa (PrPcore #2^14^) was observed in H-BSE and BSE-SW (Fig. 1d). On the other hand, no difference was observed in the glycoprofiles of BSE-SW, H-BSE, and C-BSE, as detected by mAb 6H4 (Fig. 1e). These biochemical properties of BSE-SW were maintained in the subsequent passages (data not shown).

### Neuropathological examination

**Figure 2:**
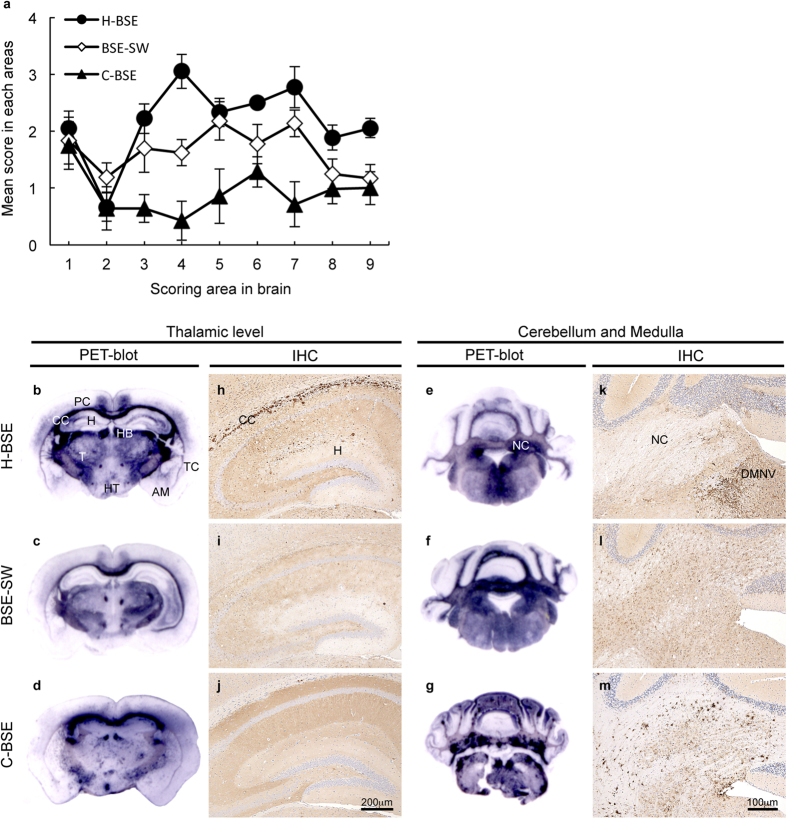
Neuropathological analysis of TgBoPrP mice. Lesion profiles (**a**), neuroanatomical distribution of PrP^Sc^ detected by PET-blot (**b–g**), and PrP^Sc^ staining types assessed by IHC (**h–m**) in the brains of BSE prion-affected TgBoPrP mice. Vacuolation in the following brain regions was scored, on a scale of 0–4 (mean values): 1, dorsal medulla; 2, cerebellar cortex; 3, superior cortex; 4, hypothalamus; 5, thalamus; 6, hippocampus; 7, septal nuclei; 8, cerebral cortex at the level of the hypothalamus and thalamus; and 9, cerebral cortex at the level of the caudate nuclei. The data are presented as mean ± standard deviation (n = 7). Black circles, H-BSE; Diamonds, BSE-SW; Black triangles, C-BSE. The degree of vacuolation in the brain of BSE-SW was different from H-BSE and C-BSE (**a**). PET blots with mAb SAF84 corresponding to the brain areas at the level of thalamus (**b–d**), and the level of medulla and cerebellum (**e–g**) are shown. IHC was performed with mAb F99/97.6.1. CC, corpus callosum; HB, habenular nucleus; PC, parietal cortex; TC, temporal cortex; H, hippocampus; T, thalamus; HT, hypothalamus; AM, amygdala; DMNV, dorsal motor nucleus of vagus nerve; NC, deep nuclei of the cerebellum. PrP^Sc^ deposits and distribution patterns in the BSE-SW brain were distinct from H-BSE and C-BSE.

The degree of brain vacuolation and neuroanatomical distribution patterns of PrP^Sc^ in TgBoPrP mice with BSE-SW were different from H-BSE and C-BSE (Fig. 2). The lesion scores of BSE-SW were lower than H-BSE scores in most brain areas, with the exception of dorsal medulla, cerebellar cortex, and thalamus (Fig. 2a). Regarding the PrP^Sc^ deposition and distribution patterns in BSE-SW, punctuate and granular PrP^Sc^ deposition was observed in the septal nucleus, corpus callosum, habenular nucleus, hypothalamus, superior colliculus, mesencephalic tegmentum, vestibular nucleus, cochlear nucleus, and reticular formation of the brainstem. Relatively heavy deposition of PrP^Sc^, consisting mainly of intraglial and intraneuronal staining, was conspicuous in the white matter of the cerebellum of BSE-SW in comparison to H-BSE (Fig. 2e,f,k,l). The PrP^Sc^ staining was minimal in the cerebral and cerebellar cortex (Fig. 2c). PrP-plaques in the subcallosal region were less frequent in BSE-SW than in H-BSE (Fig. 2h,i). In contrast, the most conspicuous feature of PrP^Sc^ staining type in TgBoPrP mice infected with H-BSE comprised heavily deposited plaques and/or large aggregates, which mainly located in the subcallosal and adjacent periventricular area of the brain (Fig. 2h). Such plaque deposits were occasionally detected in the cerebral and cerebellar cortex, but were not present in the thalamus or the brainstem. The plaques displayed birefringence with Congo red under polarized light (data not shown). The minimal PrP^Sc^ staining in the cerebral and cerebellar cortex was similar to BSE-SW (Fig. 2b). Furthermore, the PrP^Sc^ staining pattern in C-BSE was completely different from that in BSE-SW (Fig. 2d,j,g,m). These results indicate that neuropathological properties of BSE-SW in TgBoPrP mice were apparently distinct from those of H-BSE and C-BSE.

### Conformational stability studies

**Figure 3:**
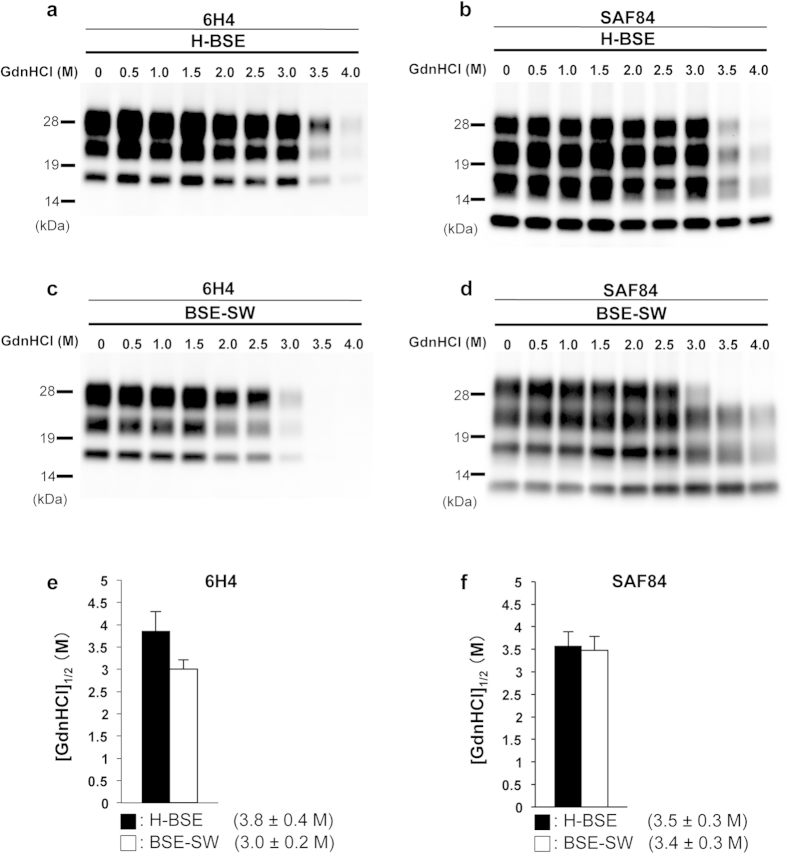
Conformational stability assay. The conformation stability of PrP^Sc^ from BSE-SW and H-BSE. GdnHCl was added at the indicated concentrations and the samples subjected to PK digestion (20 μg/ml). PrP^Sc^ was detected with mAbs 6H4 and SAF84. Molecular weights are shown on the left (kDa). [GdnHCl]_1/2_ concentration (M) was calculated based on denaturation curves obtained from densitometric analysis of western blot data. The results are mean ± standard deviation from five experiments. The black and white bars indicate H-BSE and BSE-SW, respectively.

MAbs 6H4 and SAF84 were used to conduct conformation stability studies. Western blotting with mAb 6H4 showed that the [GdnHCl]_1/2_ values (see Methods Section) for PrP^Sc^ denaturation in the H-BSE and BSE-SW brains were 3.8 ± 0.4 M and 3.0 ± 0.2 M, respectively (Fig. 3e). This analysis detected PrPcore #1 signal of 17–27 kDa from BSE-SW, and 19–29 kDa from H-BSE (Fig. 3a,c). PrPcore #1 from BSE-SW was more sensitive to GdnHCl treatments than that of H-BSE. A different result was observed with mAb SAF84, which detected both PrPcore #1 and PrPcore #2 (Fig. 3b,d). [GdnHCl]_1/2_ values of H-BSE and BSE-SW were 3.5 ± 0.3 M and 3.4 ± 0.3 M, respectively (Fig. 3f). These values were not significantly different. Signal intensity of the diglycosylated PrPcore #1 of BSE-SW was weaker than that of H-BSE during 3.0–3.5 M treatment (Fig. 3d). This result was consistent with the mAb 6H4 experiment (Fig. 3c). However, the lower three bands of PrPcore #2 showed similar signal intensities during 3.5–4.0 M treatment (Fig. 3b,d). This revealed that the conformational stability of PrPcore #1, but not PrPcore #2, is different in PrP^Sc^ from H-BSE and BSE-SW.

### Transmission of BSE-SW to cattle

To examine whether the BSE-SW prion could become a threat as a novel prion disease in cattle, three Holstein calves were challenged intracerebrally. All the inoculated animals developed progressive neurological disease. The animals exhibited initial clinical signs of the disease between 11.5 and 12.5 months post-inoculation (mpi), which included disturbance, mild fear or anxiety, mild gait changes, and, occasionally, low head carriage. After one to two months of the initial clinical signs, the animals were leaning towards the floor and rested their heads against the wall, which was followed by ataxia of the hind limbs that progressed to difficulty in getting up without assistance at the clinically terminal stage of the disease. The bodily condition gradually worsened because of a loss of weight during the two to three month clinical duration. None of the animals exhibited anorexia, nervousness, or aggression, and responded to visual, acoustic, and tactile stimuli throughout the course of the disease. The cattle were eventually culled at 13.3 mpi, 15 mpi, and 16.2 mpi before astasia, in accordance with the welfare guidelines for animal experiments. The incubation periods of cattle infected with BSE-SW (14.8 ± 1.5 mpi) were shorter than those for H-BSE, C-BSE, and L-BSE (Table 2). Obex samples from these cattle were subjected to routine BSE confirmatory tests.

### Histopathology and PrP^Sc^ immunohistochemistry of the obex of BSE-SW-affected cattle

**Figure 4:**
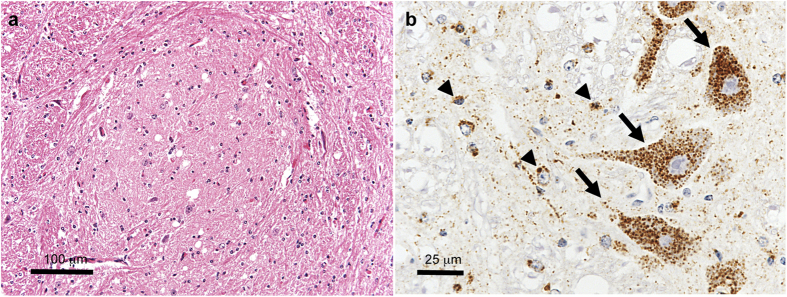
Neuropathological analysis of the obex of cattle with BSE-SW prions. (**a**) Vacuolar degeneration in the solitary nucleus of the obex level in an HE section. (**b**) Intraglial (arrowheads) and intraneuronal (arrows) PrP^Sc^ deposits stained with mAb F99/97.6.1. Size bars: a, 100 μm; b, 25 μm.

Mild vacuolation of the extracellular neuropil was observed in the dorsal motor nucleus of the vagus nerve (DMNV), the solitary nucleus, the nucleus of trigeminal nerve spinal tract, and the olivary nucleus in all animals. No intraneuronal vacuolation was seen. Spongy changes were not prominent in the gray matter of medulla oblongata at the obex (Fig. 4a). Immunolabeling of PrP^Sc^ with mAb F99/97.6.1 resulted in intraneuronal and intraglial patterns throughout the obex (Fig. 4b). Intraneuronal labeling was less common in DMNV and the hypoglossal nucleus compared to other nuclei. Fine and coarse granular PrP^Sc^ was sparsely distributed throughout the neuropil of reticular formation. No other extracellular types of PrP^Sc^, such as plaque-like and stellate deposits, were identified in the obex region.

### Molecular features of PrPcore of BSE-SW-affected cattle

**Figure 5:**
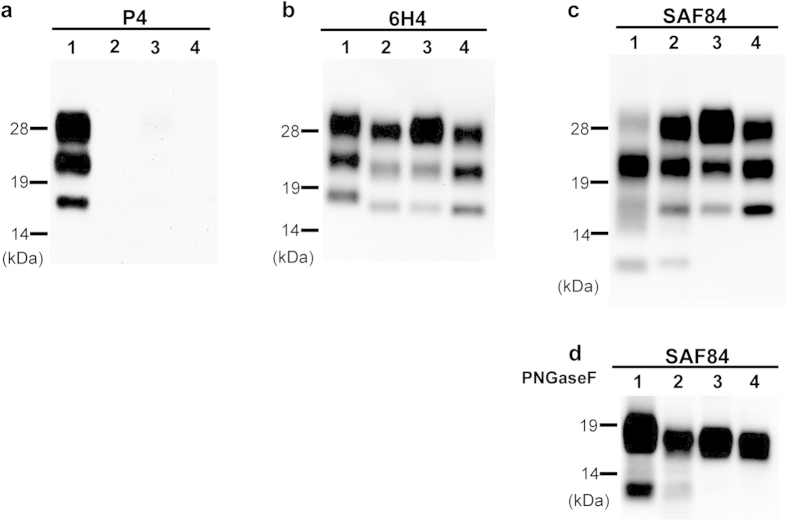
Western blot analysis of PrPcore from BSE-SW-affected cattle. PrPcore from the affected cattle were detected using mAbs: P4 (**a**), 6H4 (**b**), and SAF84 (**c**). Lane 1, H-BSE; Lane 2, BSE-SW; Lane 3, C-BSE; Lane 4, L-BSE. All the samples were digested with 40 μg/ml PK at 37 °C for 1 h. Digested aliquots were treated with PNGaseF, and were probed using mAb SAF84 (**d**). Equivalents of 0.5 mg brain tissues were loaded. Molecular markers are shown on the left (kDa).

Western blot analysis detected PrP^Sc^ from the obex tissue of the challenged cattle. The molecular features of PrPcore of BSE-SW-affected cattle were distinctly different from C-BSE, L-BSE, and H-BSE (Fig. 5). The molecular mass of PrPcore #1 of BSE-SW, as determined by mAb 6H4, was lower than H-BSE and similar to C-BSE (Fig. 5b). MAb P4 did not detect PrPcore of BSE-SW (Fig. 5a). PrPcore #2 was also observed in BSE-SW (Fig. 5c,d). These results revealed that the biochemical properties of BSE-SW have indeed been transmitted to cattle.

## Discussion

**Figure 6:**
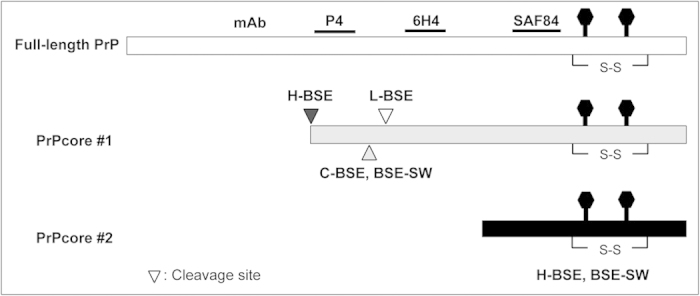
Schematic representation of PrPcore of BSE prions. Putative PK cleavage sites and PrPcore are shown, as estimated by immunoreactivity with mAbs P4, 6H4, and SAF84. PrPcore #1 is an unglycosylated PrPcore with a molecular weight of 17–19 kDa. PrPcore #2 is an unglycosylated PrPcore with a molecular weight of 12 kDa.

Our previous reports have revealed the usefulness of TgBoPrP mice for characterizing BSE prions^16^,^21^,^22^. In this study, a novel BSE, BSE-SW, was detected using this mouse model. The incubation periods of H-BSE, L-BSE, and C-BSE prions in the TgBoPrP mice were approximately 215 days, 150 days, and 190 days, respectively^16^,^21^,^22^. The BSE-SW prion showed the shortest incubation period (approximately 90 days) among the known BSE prions. We have previously performed several transmission experiments of sheep scrapie to TgBoPrP mice, but their incubation periods were over 170 days^23^, and we have not observed any prions with ~90-day incubation periods in these mice. In addition, the biochemical and biological properties of PrP^Sc^ from BSE-SW were clearly different from C-BSE, L-BSE, H-BSE, and sheep scrapie (data not shown). PrP^Sc^ of BSE-SW has some similarity to H-BSE on the account of the presence of truncated 12-kDa fragments (PrPcore #2). Fig. 6 shows the putative PK digestion site of PrP^Sc^ from BSE-SW, as assessed by immunoreactivity with mAbs P4, 6H4, and SAF84. These results argue against the possibility that the BSE-SW prion resulted from a contamination of other laboratory prion strains.

It is known that sheep scrapie comprises different prion strains^24^,^25^, and some affected sheep harbor these mixed scrapie prion strains^3^. Numerous scrapie strains had emerged in the course of several passage histories^26^. We have also previously isolated distinct scrapie strains from the brain of a scrapie-affected sheep after primary passage in wild type mice^27^. The different scrapie prion strains appeared after primary passage in sheep scrapie cases, which was considered to be due to prion strain selection. For H-BSE prions, French and Polish cases were reported, which transformed into a C-BSE-like phenotype during mouse passages^18^,^19^,^20^, revealing their potential heterogeneity. However, the BSE-SW prions described herein appear to have emerged by a different manner than our previous scrapie case^27^. The average incubation period of H-BSE in TgBoPrP mice was 320 days at first passage, and then shortened to 227 days and 216 days at secondary and third passages, respectively. Based on this observation, H-BSE prions were adapted to TgBoPrP mice at second passage. By the third passage, no multiple prion strains in the H-BSE material were evident. BSE-SW prion with a shorter incubation period emerged after H-BSE has adapted to TgBoPrP mice. The results of this study suggest that BSE-SW emerged through a conformational rearrangement. Alternatively, the BSE-SW prion was only present in a very small titer in H-BSE and required several passages in sensitive animals to emerge after selection from a mixture of preexisting prion strains. The Canadian H-BSE sample that was used in this study was also used to inoculate wild type mice, but we did not observe the emergence of a C-BSE-like phenotype in those circumstances (unpublished data). The underlying mechanisms of the emergence of new prion strains are important to elucidate prion heterogeneity. This novel BSE strain has never been observed in field BSE cases, but our experiments reveal the potential risk associated with H-BSE.

It has been suggested that different conformations of PrP^Sc^ are involved in the prion strain diversity^28^, and that rearrangement of PrP^Sc^ from a uniform conformation causes the emergence of new host-adapted PrP^Sc ^3. PrP^Sc^ of BSE-SW exhibited different conformational stability from H-BSE. It is also known that strain “mutation or transformation” may occur upon intraspecies transmission, where the PrP amino acid sequences of the host and the donor are identical^3^. These finding are consistent with the conclusion that PrP^Sc^ of BSE-SW has a different conformation than H-BSE.

Furthermore, our new strain was successfully transmitted to cattle. Standard diagnostic testing for BSE confirmed the presence of spongiform changes associated with PrP^Sc^ accumulation in the obex, and the challenged cattle fulfilled BSE criteria (Figs 4 and 5). The disease phenotype and features of PrP^Sc^, different from the known types of BSE, indicated that this prion could cause a novel type of atypical BSE. The shorter incubation periods in cattle were consistent with the relative incubation periods in TgBoPrP mice, and indicate high virulence of this novel prion. Further analysis of diseased cattle is necessary to clarify its characteristics. Such studies could help to elucidate the mechanisms of conformational change in PrP^Sc^, which lead to the propagation of new prion strains.

The ban on meat-and-bone meal in livestock feed has contributed to the decline in C-BSE occurrences^6^. Recently, easing of the BSE-related regulations and control measures has been discussed. The origin of atypical BSE remains unknown, but it has been proposed to be spontaneous or sporadic^29^. H-BSE has been reported to transform into a C-BSE-like phenotype during animal passages^18^,^19^,^20^. Furthermore, we have shown here that the sequential transmission of H-BSE in TgBoPrP mice, i.e., to a homologous bovine PrP context, generated a novel type of BSE. Considering these observations, a continuous feed ban program may be necessary even after C-BSE is eradicated. Prohibiting the recycling of spontaneously occurring H-BSE prions in cattle should help to prevent both re-emerging and emerging types of BSEs.

## Methods

### Ethics statement

Procedures involving animals have been approved by the Animal Care and Use Committee at the National Institute of Animal Health (approval ID: 11-008, 13-005). Animal experiments were performed in accordance with the Guidelines for Animal Transmissible Spongiform Encephalopathy Experiments of the Ministry of Agriculture, Forestry, and Fisheries of Japan.

### Transgenic mice

TgBoPrP mice overexpressing the bovine *PrP* gene (encoding BoPrP) in a mouse *PrP* deficient background were used. These mice harbored the cattle *PrP* gene containing six copies of the octarepeat sequence (EMBL, X55882), and produced approximately eight times more BoPrP per gram of protein than found in the cattle brain^30^.

### BSE material

Brain samples of Canadian H-BSE cattle, courteously provided by Dr. S. Czub, were used in this study^31^. Mouse-passaged L-BSE and C-BSE prions were also used. These prions were routinely maintained by serial passaging into TgBoPrP mice, as described previously^21^,^22^. C-BSE and L-BSE prions were adapted to TgBoPrP mice by serial passaging, and their incubation periods were approximately 190 days and 150 days, respectively. Brain samples from C-BSE, L-BSE, and H-BSE-affected cattle were also used^10^,^32^,^33^.

### Mouse transmission study

Brain tissues from BSE-affected animals were homogenized in nine volumes of phosphate buffered saline (PBS) using a multi-bead shocker (Yasui Kikai) and centrifuged at 1,000 × *g* for 5 min at room temperature (RT). Three-week-old female TgBoPrP mice were inoculated intracerebrally with 20 μl supernatant. Following inoculation, clinical status of the mice was monitored daily to assess the onset of neurological signs. The brains of diseased mice were removed and stored at -80 °C for biochemical analysis or fixed for histopathology. For the fourth passage, eight diseased mice brains from the third passage were used to independently challenge to TgBoPrP mice.

### Cattle transmission study

Three female 3-4-month-old Holstein calves were challenged intracerebrally with 1 ml of 10% brain homogenate of BSE-SW-affected TgBoPrP mice, as described previously^10^. Animals were euthanized before ataxia deterioration. The brains of diseased cattle were removed and stored at −80 °C for western blotting analysis or fixed for pathological examination (i.e., BSE confirmatory tests).

### Extraction of PrP^Sc^ from BSE-affected TgBoPrP mice and cattle

Brain tissues were homogenized in PBS (20% homogenate, w/v) using a multi-bead shocker. The brain homogenate (125 μl) was mixed with an equal volume of buffer containing 4% (w/v) Zwittergent 3–14 (Calbiochem), 1% (w/v) Sarkosyl, 100 mM NaCl, and 50 mM Tris-HCl (pH 7.6), and incubated with 6.25 μl of 40 mg/ml collagenase solution. The samples (50 μg/ml for mice brain tissues, and 40 μg/ml for cattle brain tissues) were then subjected to PK (Roche Diagnostic) digestion at 37 °C for 1 h to detect PK-resistant PrP^Sc^ fragments (PrPcore). PK digestion was terminated with 2 mM 4-(2-aminoethyl)benzenesulfonyl fluoride hydrochloride (Pefabloc; Roche Diagnostic). The samples were mixed with equal volumes of 2-butanol:methanol mixture (5:1) and centrifuged at 20,000 × *g* for 10 min. The pellets were resuspended in gel-loading buffer containing 2% (w/v) SDS, and were then boiled for 10 min before western blotting. After PK treatment, some samples were deglycosylated with N-glycosidase F (PNGaseF; New England Biolabs), following the manufacturer’s instruction.

### Antibodies

The following monoclonal antibodies (mAbs) against PrP were used in this study: P4 (R-Biopharm), 6H4 (Prionics), SAF84 (SPI-bio), and F99/97.6.1 (VMRD). MAb P4 recognizes amino acid residues 101–107 of bovine PrP sequence^34^. MAbs 6H4 and SAF84 recognize amino acid residues 156–163^35^ and 175–180^36^, respectively, of the bovine PrP sequence. F99/97.6.1 recognizes C-terminus of PrP, amino acid residues 229–232^10^.

### Western blot analysis

Samples were separated by SDS-PAGE and blotted electrically onto a PVDF membrane (Millipore). The blotted membrane was incubated with mAbs P4, 6H4, and SAF84 at RT for 1 h. MAb binding was detected by horseradish peroxidase-conjugated anti-mouse IgG. Signals were developed with a chemiluminescent substrate (SuperSignal; Pierce Biotechnology).

### Neuropathology, immunohistochemistry, and PET-blot analysis

Histopathological analysis of TgBoPrP mice and cattle was performed according to a previously described method^10^,^16^,^21^. Briefly, the brains were fixed in 10% buffered formalin solution (pH 7.4) containing 10% methanol. The formalin-fixed brains were immersed in 98% formic acid, and embedded in paraffin wax. Sections (4 μm thick) were cut and stained with hematoxylin and eosin (HE). The lesion profile was determined by scoring the vacuolar changes in nine standard grey matter areas, as previous described^37^. For PrP^Sc^ immunohistochemistry (IHC), sections were incubated with mAbs SAF84 or F99/97.61, followed by incubation with anti-mouse universal immunoperoxidase polymer (Histofine Simple Stain MAX-PO (M), Nichirei) as the secondary antibody, and visualized using 3,3′-diaminobenzidine tetrachloride as the chromogen. Finally, the sections were counterstained with hematoxylin. Paraffin embedded tissue (PET) blot was performed as described previously^21^. Dewaxed membranes were treated with 80 μg/ml of PK for 30 min at 37 °C, and then denatured using 3 M guanidine thiocyanate for 10 min at RT. The blotted membranes were incubated with mAb SAF84 for 90 min at RT. Signals were detected using Histofine MAX-AP (M) Kit (Nichirei) with 5-bromo 4-chloro-3-indolyl phosphate and nitro blue tetrazolium (BCIP/NBT; Roche Diagnostics) as substrates.

### Conformational stability assay

Conformation stability assay was performed according to a previously described method with minor modification^38^. Briefly, 50 μl of 10% brain homogenate were added to an equal volume of guanidine hydrochloride (GdnHCl), concentration range 0-8 M. Mixed samples were incubated at 37 °C for 1 h. The samples were diluted by the addition of 850 μl Tris buffer containing 10 mM Tris-HCl (pH 8.0), 150 mM NaCl, 0.5% Triton-X, and 0.5% deoxycholate. Following this, 50 μL GdnHCl were added to each sample to obtain 0.4 M final concentration. Next, the samples were digested with 20 μg/ml PK at 37 °C for 1 h. PrP^Sc^ concentration and western blot analysis were carried out as described above. Conformational stability was examined using mAbs 6H4 and SAF84. Denaturation curves were obtained by densitometric analysis using Fluorochem software (Alpha Innotech Co.). GdnHCl concentration at half maximal denaturation ([GdnHCl]_1/2_) was used as a measure of the relative conformational stability of PrP^Sc^. [GdnHCl]_1/2_ was calculated based on the denaturation curves.

## Additional Information

**How to cite this article**: Masujin, K. *et al*. Emergence of a novel bovine spongiform encephalopathy (BSE) prion from an atypical H-type BSE. *Sci. Rep.* **6**, 22753; doi: 10.1038/srep22753 (2016).

## Figures and Tables

**Table 1:** Transmission of H-BSE in TgBoPrP mice.

Passage history					Remarks
First	Second	Third	Fourth	Fifth	
320.1 ± 12.2^a^ (10/10)^b^ [318^c^]	226.9 ± 4.2 (7/7) [230]	215.6 ± 5.0 (40/40)			H-BSE
		#5^d^ [207]	220.8 ± 0.3 (7/7)		
		#1 [209]	214.0 ± 10.8 (5/5)		
		#6 [213]	209.6 ± 0.8 (6/6)		
		#8 [215]	223.3 ± 7.8 (4/4)		
		#2 [216]	220.0 ± 4.4 (6/6) [234]	216.7 ± 3.0 (12/12)	
		#7 [216]	210.3 ± 1.7 (6/6)		
		#4 [228]	225.0 ± 0 (6/6)		
		#3 [221]	108.8 ± 4.0 (20/20) [106]	97.3 ± 3.7 (10/10)	BSE-SW

^a^Average ± standard deviation (days).

^b^Numbers of affected/inoculated mice are shown in parentheses.

^c^Incubation periods of mice used for subsequent passages are shown in brackets.

^d^Experimental group number at fourth passage: each experiment used a different brain sample from affected mice at third passage.

**Table 2:** Intracerebral challenge of BSE prions in cattle.

	BSE-SW	C-BSE	L-BSE	H-BSE
Incubation periods	14.8 ± 1.5^a^	22.5 ± 1.9	16.2 ± 0.4	18.7 ± 1.6
Clinical signs	Ataxia, Anxiety	Ataxia, Hyperesthesia	Ataxia, Anxiety	Ataxia, Anxiety, Myoclonus
WB (obex)	+	+	+	+
IHC (obex)	+	+	+	+
Reference	This study	[33]	[32]	[10]

^a^Average ± standard deviation (months).

Abbreviations: WB, western blotting; IHC, immunohistochemistry; +, positive for PrP^Sc^. Obex, the diagnosis region of the central nervous system.
